# Curcumin ameliorates macrophage infiltration by inhibiting NF-κB activation and proinflammatory cytokines in streptozotocin induced-diabetic nephropathy

**DOI:** 10.1186/1743-7075-8-35

**Published:** 2011-06-10

**Authors:** Vivian Soetikno, Flori R Sari, Punniyakoti T Veeraveedu, Rajarajan A Thandavarayan, Meilei Harima, Vijayakumar Sukumaran, Arun Prasath Lakshmanan, Kenji Suzuki, Hiroshi Kawachi, Kenichi Watanabe

**Affiliations:** 1 Department of Clinical Pharmacology, Faculty of Pharmaceutical Sciences, Niigata University of Pharmacy and Applied Life Sciences, Niigata City, Japan; 2 Department of Pharmacology, Faculty of Medicine, University of Indonesia, Jakarta, Indonesia; 3 Department of Gastroenterology and Hepatology, Niigata University Graduate School of Medical and Dental Sciences, Niigata, Japan; 4 Department of Cell Biology, Institute of Nephrology, Niigata University Graduate School of Medical and Dental Sciences, Niigata, Japan

## Abstract

**Background:**

Chronic inflammation plays an important role in the progression of diabetic nephropathy (DN) and that the infiltration of macrophages in glomerulus has been implicated in the development of glomerular injury. We hypothesized that the plant polyphenolic compound curcumin, which is known to exert potent anti-inflammatory effect, would ameliorate macrophage infiltration in streptozotocin (STZ)-induced diabetic rats.

**Methods:**

Diabetes was induced with STZ (55 mg/kg) by intraperitoneal injection in rats. Three weeks after STZ injection, rats were divided into three groups, namely, control, diabetic, and diabetic treated with curcumin at 100 mg/kg/day, p.o., for 8 weeks. The rats were sacrificed 11 weeks after induction of diabetes. The excised kidney was used to assess macrophage infiltration and expression of various inflammatory markers.

**Results:**

At 11 weeks after STZ injection, diabetic rats exhibited renal dysfunction, as evidenced by reduced creatinine clearance, increased blood glucose, blood urea nitrogen and proteinuria, along with marked reduction in the body weight. All of these abnormalities were significantly reversed by curcumin. Hyperglycemia induced the degradation of IκBα and NF-κB activation and as a result increased infiltration of macrophages (52%) as well as increased proinflammatory cytokines: TNF-α and IL-1β. Curcumin treatment significantly reduced macrophage infiltration in the kidneys of diabetic rats, suppressed the expression of above proinflammatory cytokines and degradation of IκBα. In addition, curcumin treatment also markedly decreased ICAM-1, MCP-1 and TGF-β_1 _protein expression. Moreover, at nuclear level curcumin inhibited the NF-κB activity.

**Conclusion:**

Our results suggested that curcumin treatment protect against the development of DN in rats by reducing macrophage infiltration through the inhibition of NF-κB activation in STZ-induced diabetic rats.

## Background

Diabetic nephropathy (DN) is the largest single cause of end-stage renal failure worldwide. In spite of the available modern therapies of glycaemic and blood pressure control, many patients continue to show progressive renal damage [1]. Therefore, it is extremely important to identify novel interventions to halt the progression of DN.

Recently, DN has been considered as an inflammatory disease [2], in which macrophage infiltration into glomeruli is associated with the progression of glomerular injury [3]. Monocytes/macrophages are the principle inflammatory cells found in the diabetic kidney [3-5], these cells extravasculated from the blood-stream and attracted to the target tissue through a process mediated by various chemokines secreted from resident glomerular cells such as monocyte chemotactic protein (MCP)-1 and adhesion molecules such as intercellular adhesion molecule (ICAM)-1 [6-8]. MCP-1 is a potent chemokine that is known to influence both macrophage accumulation and macrophage function and its expression increases gradually in diabetic kidneys in animal models [9,10]. Renal expression of ICAM-1, a 90-kD cell surface glycoprotein that plays a major role in the regulation of interactions with immune cells whose expression is upregulated at the sites of inflammation, is known to be increased in experimental type 1 diabetic animals. Furthermore, macrophage infiltration was found to be blocked by anti-ICAM-1 antibody, confirming that ICAM-1 mediates macrophage infiltration into diabetic kidney [11]. These findings suggest that ICAM-1 and MCP-1 may play an important role in the pathogenesis of DN via the induction of inflammatory cell infiltration.

Transcription factors such as nuclear factor-κB (NF-κB) regulate the gene expression of several cytokines, chemotactic and matrix proteins involved in inflammation, immunological responses, and cell proliferation [12]. Studies have demonstrated that high glucose-induced NF-κB activation in mesangial cells may play a role in diabetic renal injury through upregulation of ICAM-1 protein and mRNA expression in rat mesangial cells [13,14]. In addition, activated NF-κB shows an important role in the upregulation of MCP-1 in human DN [15]. NF-κB is also known to activate its downstream inflammatory mediators such as transforming growth factor (TGF)-β_1_, inducible nitric oxide synthase (iNOS), fibronectin, tumor necrosis factor (TNF)-α and interleukin (IL)-1β [14,16].

Curcumin is the active ingredient in the traditional herbal remedy and dietary spice turmeric (*Curcuma longa*) and is the subject of clinical trials for various diseases such as cancer, Alzheimer's disease, and ulcerative colitis [17]. The polyphenol curcumin (diferuloylmethane) comprises 2-8% of most turmeric preparations and is generally regarded as its most active component, having potent antioxidant, anti-inflammatory and anticarcinogenic properties. Modern science has revealed that curcumin mediates its effects by modulation of several important molecular targets, including transcription factors (e.g., NF-κB, activator protein (AP)-1, Early growth response gene (Egr)-1, β-catenin and Peroxisome proliferator-activated receptor (PPAR)-γ), enzymes (e.g., cyclooxigenase 2 (COX2), 5-lipoxygenase (LOX), iNOS and hemeoxygenase-1 (HO)-1), cell cycle proteins (e.g., cyclin D1 and p21), cytokines (e.g., TNF-α, IL-1, IL-6 and chemokines), receptors (e.g., Epidermal Growth Factor Receptor (EGFR) and Human Epidermal growth factor Receptor2 (HER2)) and cell surface adhesion molecules. Because it can modulate the expression of these targets, curcumin is now being used to treat cancer, arthritis, diabetes, Crohn's disease, cardiovascular diseases, osteoporosis, Alzheimer's disease, psoriasis and other pathologies [18].

Several in vitro and in vivo studies have demonstrated that curcumin inhibits activation of NF-κB proinflammatory signaling pathways and reduces the influx of macrophages [19-21]. However, to the best of our knowledge, studies have not been revealed the effect of curcumin on macrophages accumulation in DN. In the present study, we investigated the effect of curcumin on macrophages accumulation and the expression of ICAM-1, MCP-1 and TGF-β_1 _in experimental diabetic kidney. In addition, the relationship between the extent of transcription factor NF-κB and proinflammatory cytokines, TNF-α and IL-1β, expression in renal tissue was elucidated.

## Methods

### Animals and experimental protocol

All animal studies were treated in accordance with the guidelines for animal experimentation of our institute [22]. Male Sprague-Dawley rats (Charles River Japan Inc., Kanagawa, Japan), weighing approximately 250-300 g (8 weeks of age), were randomly divided into three groups (*n *= 5): non-diabetic normal animals used as a controls (C), diabetic animals treated with vehicle 1% gum Arabic (D), and diabetic animals treated with curcumin 100 mg/kg body weight [23] diluted in vehicle 1% gum Arabic (Cur). Diabetes was induced by a single intraperitoneal injection of streptozotocin (STZ 55 mg/kg body weight in 20 mM citrate buffer, pH 4.5), while the control animals received 20 mM citrate buffer solution. The use of STZ to induce diabetes provides an animal model of type 1 diabetes, as the drug causes pancreatic β-islet cell apoptosis. Diabetes was confirmed by tail-vein blood glucose levels using Medi-safe chips (Terumo Inc., Tokyo, Japan), and diabetes was defined if blood glucose ≥ 300 mg/dL. Curcumin was started at three weeks after STZ injection and was administered via oral gavage for 8 weeks. Rats were housed in a temperature-controlled room and were given free access to water and standard laboratory chow during the study period. All rats were sacrificed at 11 weeks after the induction of diabetes for analysis of renal tissue.

### Biochemical analysis

Each week, rats were weighed and their blood glucose levels were measured. Urine samples were collected over a 24 h period in individual metabolic cages for the measurement of protein in urine at 1, 3, 7, and 11 weeks and were determined by the Bradford method. Urine creatinine was determined by the Jaffe method at 11 week. At the end of experimentation, heparinized whole blood was collected from anesthetized rats *via *heart puncture. EDTA-blood was centrifuged at 3000 *g*, 15 min at 4°C for the separation of plasma. The plasma was used for the estimation of blood urea nitrogen (BUN) and creatinine. Creatinine clearance was calculated in individual rats as follows: Creatinine clearance = urine creatinine × urine volume/plasma creatinine × time [24].

### Chemicals

Unless otherwise stated all reagents were of analytical grade and were purchased from Sigma-Aldrich (Tokyo, Japan).

### Histopathological analysis

The kidney was decapsulated. Half of the kidney was immediately snap-frozen in liquid nitrogen for subsequent protein extraction and enzymatic assays. The remaining excised kidneys were cut into about 2-mm thick transverse slices and fixed in 10% formalin. After being embedded in paraffin, several transverse sections were obtained from the kidney and stained with Periodic acid-Schiff (PAS) for histological evaluation. The frequency and severity of lesions in kidney were assessed semi-quantitatively by light microscopy. Forty glomeruli in each kidney were graded in accordance with their severity of glomerular damage (0, no lesion; 1, sclerosis of < 25% of the glomerulus; 2, sclerosis of 26-50%; 3, sclerosis of 51-75%; and 4, sclerosis of > 75% of the glomerulus). The glomerulosclerosis indexes were calculated using the following formula, (glomerulosclerosis index = (1 × n_1_) + (2 × n_2_) + (3 × n_3_) + (4 × n_4_)/n_0 _+ n_1 _+ n_2 _+ n_3 _+ n_4_, where n_x _is number of glomeruli in each grade of glomerulosclerosis) as reported previously [25].

### Immunohistochemistry

Formalin-fixed, paraffin-embedded kidney tissue sections were used for immunohistochemical staining. After deparaffinization and hydration, the slides were washed in Tris-buffered saline (TBS; 10 mM/L Tris HCl, 0.85% NaCl, pH 7.2). Endogenous peroxidase activity was quenched by incubating with the slides in methanol and 0.3% H_2_O_2 _in methanol. After overnight incubation with the primary antibody, that is, mouse monoclonal anti-ED1 antibody (diluted 1:50) (sc-59103; Santa Cruz Biotechnology Inc. CA, USA) at 4°C, the slides were washed in TBS and horseradish peroxidase (HRP)-conjugated goat antimouse secondary antibody was then added and the slides were further incubated at room temperature for 1 h. The slides were washed in TBS and incubated with diaminobenzidine tetrahydrochloride as the substrate, and counterstained with hematoxylin. A negative control without primary antibody was included in the experiment to verify the antibody specificity. Intraglomerular ED-1-positive cells were counted in 200 glomeruli/group under 400-fold magnification and expressed as cells/glomerular cross section (gcs) [26].

### Immunofluorescence staining for ICAM-1

For immunofluorescence, tissues were fixed in 10% buffered formaldehyde solution and embedded in paraffin. Sections underwent microwave antigen retrieval, were blocked with 10% rabbit serum in 1% BSA and were then incubated with polyclonal goat anti-ICAM-1 antibody (sc-1511; Santa Cruz). Binding sites of the primary antibody were revealed with fluorescein isothiocyanate-conjugated secondary antibody. Samples were visualized with a fluorescence microscope at 400-fold magnification (CIA-102; Olympus) [27].

### Cytosolic and nuclear extract homogenization

The kidney was cut and immediately frozen in liquid nitrogen and kept at -80°C until use. The frozen kidney was ground to a powder and then mixed in ice-cold HEPES buffer (10 mM HEPES, 0.2% Triton X-100, 50 mM NaCl, 0.5 mM sucrose, 0.1 mM EDTA, protease, and phosphatase inhibitors) and homogenized with an ice-chilled Dounce homogenizer at 4°C. This was spun at 10,000 rpm for 10 min, and the supernatant was aliquoted and stored at -80°C as the cytosolic extract. The pellet was suspended in ice-cold buffer (10 mM HEPES, 500 mM NaCl, 10% glycerol, 0.1 mM EDTA, 0.1 mM EGTA, 0.1% IGEPAL, and protease and phosphatase inhibitors) and vortexed at 4°C for 15 min and centrifuged for 10 min at 14,000 rpm. The resulting supernatant was aliquoted and stored as the nuclear extract at -80°C. The absence of cross-reactivity with β-actin in Western blots confirmed the purity of nuclear extracts. A small aliquot of cytosolic and nuclear extract was kept at 4°C for protein estimation [28].

### Western blotting

Cytosol (70 μg total protein) and nuclear extracts (50 μg total protein) were separated on a 7.5-15% polyacrilamide gel and electophoretically transferred to nitrocellulose membrane. Membranes were blocked with 5% non-fat dry milk in Tris-buffered saline Tween (20 mM Tris, pH 7.6, 137 mM NaCl, and 0.1% Tween 20) for 3 h at room temperature, followed by an overnight incubation at 4°C with polyclonal antibodies to rat ICAM-1, IκBα (sc-371; Santa Cruz), TNF-α (sc-1350; Santa Cruz), IL-1β (sc-7884; Santa Cruz), NF-κB p65 (Cell Signaling #4764), p-NF-κB (Ser276) (sc-101749; Santa Cruz), TGF-β_1 _(Promega G-1221), MCP-1 (sc-1785; Santa Cruz) or β-actin (Cell Signaling). All antibodies were used at a dilution of 1:1000. Three times after washing with Tris-buffered saline Tween 20 (TBS-T), incubation with appropriate HRP-conjugated secondary antibodies were performed for 1 h at room temperature. After three additional TBST washes, the immunoreactive bands were visualized by enhanced chemiluminescence (Amersham Biosciences, Buckinghamshire, UK) according to the manufacturer's instructions. The levels of β-actin and lamin A (sc-20680; Santa Cruz) were estimated in cytosol samples and in nuclear extract samples, respectively, to check for equal loading of samples. Films were scanned and band densities were quantified with densitometric analysis using Scion Image program (Epson GT-X700, Tokyo, Japan).

### RNA extraction

Kidney tissues were preserved by immersion in RNAlater (Ambion Inc., Austin, TX) immediately after sampling. Total RNA was extracted after homogenization using Ultra TurraxT8 (IKA Labortechinik, Staufen, Germany) in TRIzol reagent (Invitrogen Corp., Carlsbad, CA) according to the standard protocol. cDNA was synthesized by reverse transcription using total RNA (2 μg) as a template (Super Script II; Invitrogen Corp).

### Gene expression analysis by real-time RT-PCR

Gene expression analysis was performed by real-time reverse transcription polymerase chain reaction (RT-PCR) (Smart cycler; Cepheid, Sunnyvale, CA) using cDNA synthesized from the diabetic speciemen. Primer sequences were as follows: IL-1β (forward), CTTCAATCTCACAGCAGCACATCTCG, (reverse), TCCACGGGCAAGACATAGGTAGC; TNF-α (forward), CCCCAAAGGGATGAGAAGTT, (reverse), CACTTGGTGGTTTGCTACGA; GAPDH (forward), GCTCATTTCCTGGTATGACAACG, (reverse), AGGGGTCTACATGGCAACTG. Real-time RT-PCR, monitoring with a TaqMan probe (TaqMan Gene expression assays; Applied Biosystems, Foster City, CA) was performed according to the following protocol: 600 seconds at 95°C, followed by thermal cycles of 15 seconds at 95°C, and 60 seconds at 60°C for extension. Relative standard curves representing several 10-fold dilutions (1:10: 100: 1000: 10,000: 100, 000) of cDNA from kidney tissue samples were used for linear regression analysis of other samples. Results were normalized to GAPDH mRNA as an internal control and are thus shown as relative mRNA levels.

### Statistical analysis

All values are expressed as means ± SEM and were analyzed using one-way analysis of variance (ANOVA) followed by Tukey's methods for post hoc analysis and two-tailed *t*-test when appropriate. A value of *p *< 0.05 was considered statistically significant. For statistical analysis, GraphPad Prism 5 software (San Diego, CA, USA) was used.

## Results

### Animal data

**Figure 1:**
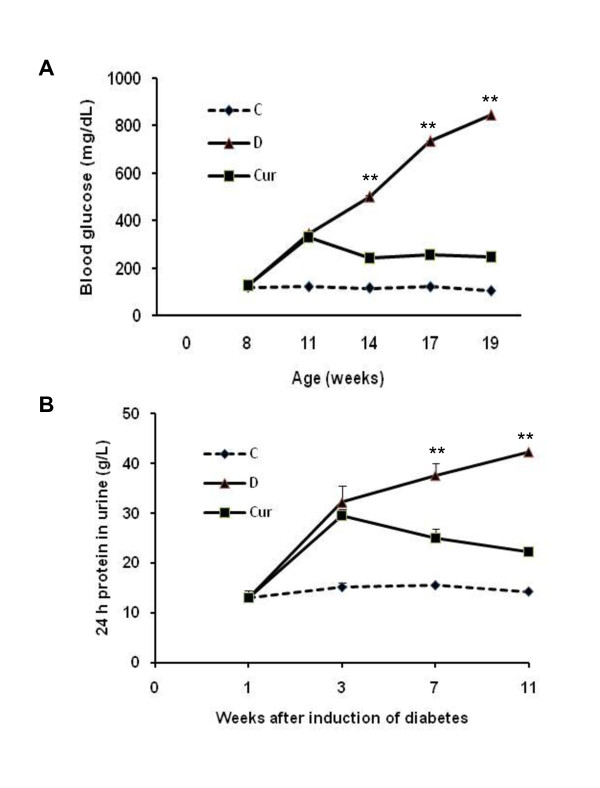
**Time-course changes in blood glucose (A) and 24 h urine protein (B)**. Blood glucose as well as 24 h urine protein increased progressively in the untreated diabetic rats following induction of diabetes. Curcumin treatment significantly reduced blood glucose and 24 h urine protein in the beginning of treatment and these were maintained throughout the study period until sacrifice. Values are means ± SEM. **p < 0.01 *vs *Curcumin.

At the end of the 11^th ^week, body weight and the ratio of kidney weight to body weight, a marker for the development of DN was significantly decreased and increased, respectively in diabetic rats, and this ratio was significantly decreased in diabetic rat-treated with curcumin. Diabetic rats also exhibited increased plasma creatinine, increased BUN, and decreased creatinine clearance (CCr). All of these abnormalities were significantly decreased by curcumin treatment (Table 1). Treatment with curcumin also prevented body weight loss in diabetic rats, but this effect was not significant compared with that of untreated diabetic rats. During the study period, plasma glucose (Figure 1A) and 24 h urinary protein excretion (Figure 1B) increased progressively in diabetic rats which was significantly abrogated by the administration of curcumin (*p *< 0.05).

**Table 1:** Biochemical parameters in experimental animals at 11 weeks

	C (*n *= 5)	D (*n *= 5)	Cur (*n *= 5)
Body weight (BW) (g)	539.5 ± 19.24	325.8 ± 15.88*	341.5 ± 15.11
Kidney weight (KW) (g)	1.5 ± 0.04	1.94 ± 0.11*	1.65 ± 0.11
KW/BW ratio × 1000	2.78 ± 0.05	5.94 ± 0.12*	4.83 ± 0.19^#^
Plasma creatinine (mg/dL)	0.55 ± 0.02	1.06 ± 0.13*	0.69 ± 0.03^#^
CCr (mL/min)	3.8 ± 0.9	0.81 ± 0.08*	3.61 ± 0.48^#^
BUN (mg/dL)	22.63 ± 0.95	38.05 ± 1.8*	30.58 ± 2.2^#^

BUN: blood urea nitrogen, CCr: creatinine clearance, KW/BW: ratio of kidney weight to body weight

Results are presented as the mean ± SEM

• *p < 0.05 *vs*. C

*• #p *< 0.05 *vs*. D

### Histology

**Figure 2:**
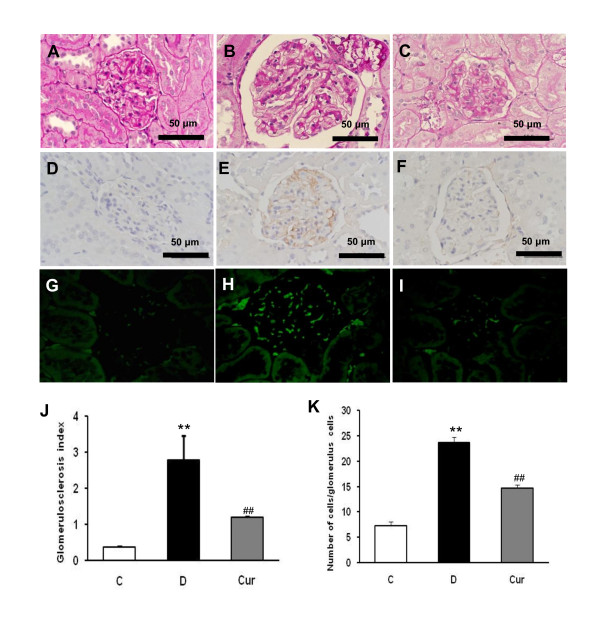
**Histological staining with Periodic acid-Schiff's (PAS) in glomeruli (A-C) shows the glomerular and tubulointerstitial structure of a normal rat kidney (A), diabetic rat kidney (B), in which significant changes in glomerular and tubulointerstitial structure were noted**. Diabetic rat developed the pathological characteristics of early diabetic nephropathy, including glomerular hypertrophy, mesangial expansion and sclerosis of glomerulus (B). (C) Diabetic rat treated with curcumin showed amelioration of sclerosis of glomerulus. D-F: immunohistochemistry staining for macrophage (ED-1-positive cells). G-I: immunofluorescence staining for ICAM-1 in glomeruli. Kidney tissues were harvested from normal rat as a control (A, D, G), diabetic rat (B, E, H) and curcumin-treated rat (C, F, I). Quantification results are shown for glomerulosclerosis index in each group (J) and number of macrophages (ED-1-positive cells) (K). Values are means ± SEM. **p < 0.05 *vs *C; ^##^*p *< 0.05 *vs *D (J). **p < 0.01 *vs *C; ^##^*p *< 0.01 *vs *D (K).

Normal histology was seen in the control rats (Figure 2A). On the other hand, histological examination of the kidney of diabetic rats had marked histological changes in the glomerular and tubular structure (Figure 2B). In the untreated diabetic rats, 64% of the glomeruli were segmentally sclerosed (Figure 2J), whereas curcumin-treated rats developed only 27.4% (*p *< 0.05 compared with untreated diabetic rats) segmental sclerosis (Figure 2C,J).

### Effect of curcumin on macrophage infiltration

Kidneys from control rats did not show any significant macrophage infiltration (Figure 2D). On the other hand, diabetic rats demonstrated prominent macrophage (ED-1-positive cells) infiltration in the glomerulus (Figure 2E,K), where as diabetic rats treated with curcumin showed marked reduction of macrophage influx by 32% (*p *< 0.01) (Figure 2F,K).

### Effect of curcumin on NF-κB and IκBα

**Figure 3:**
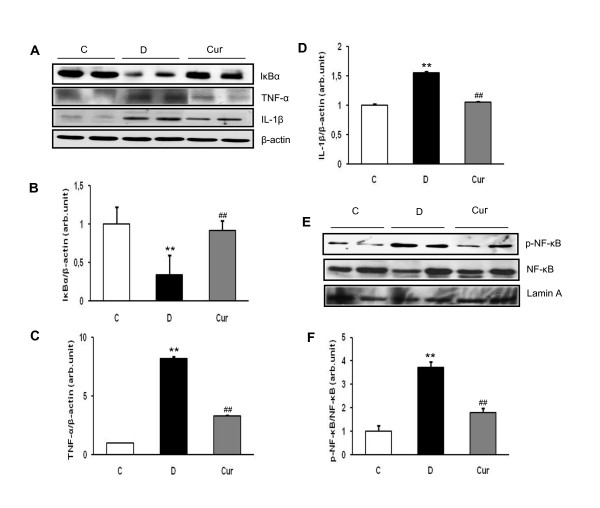
**Renal expression of IκBα, TNF-α, IL-1β, p-NF-κB and NF-κB**. (A) Representative Western blots showing specific bands for IκBα, TNF-α, IL-1β and β-actin as an internal control. Equal amounts of protein sample obtained from whole kidney homogenates were applied in each lane. These bands are representative of five separate experiments. (B-D) Graphical representation of data from Western blots analyses. The mean density values of IκBα, TNF-α and IL-1β are expressed as ratios relative to that of β-actin. Values are means ± SEM. **p < 0.05 *vs *C; ^##^*p *< 0.05 *vs *D for IκBα, **p < 0.01 *vs *C; ^##^*p *< 0.01 *vs *D for TNF-α and IL-1β. (E) Representative Western blots showing specific bands for phosphorylated NF-κB, NF-κB as an internal control and lamin A for equal loading of nuclear sample. (F) Densitometric data of protein analysis. The mean density values of p-NF-κB are expressed as ratios relative to that of NF-κB. C, age-matched normal rats; D, diabetic-treated rats administered with vehicle; Cur, diabetic rats treated with curcumin 100 mg/kg/day. ** *p *< 0.01 *vs*. C; ^## ^*p *< 0.05 *vs*. D based on one way ANOVA followed by Tukey's test.

In this study (Figure 3A,B), we observed that cytosolic IκBα in the kidney of diabetic rats was significantly lower than in the control (*p *< 0.05) and curcumin treatment significantly reduced IκBα degradation (*p *< 0.05). In addition, NF-κB was activated, which is measured by the extent of phosphorylation of its p65 regulatory subunit (p-NF-κB) in diabetic rats compared with that of controls. Curcumin treatment to the diabetic rats resulted in a mitigatory effect and showed decreased levels of activated NF-κB compared with that of diabetic rats (Figure 3E,F), in which nuclear NF-κB was 3.73 fold higher in diabetic rats than in the control (*p *< 0.01) and curcumin treatment decreases the activated NF-κB by 2.08 fold than in untreated diabetic rats (*p *< 0.05).

### Effect of curcumin on renal expression of TNF-α and IL-1β

**Figure 4:**
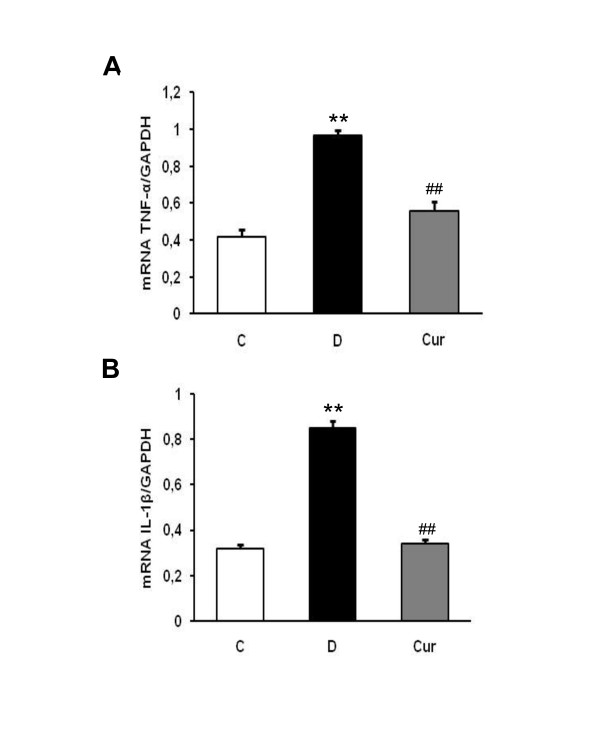
**Effect of curcumin on renal messenger RNA expression levels of TNF-α (A) and IL-1β (B) in rats with DN were determined by quantitative RT-PCR**. The expression level of each sample is expressed relative to the expression level of GAPDH gene. C, age-matched normal rats; D, diabetic-treated rats administered with vehicle; Cur, diabetic rats treated with curcumin 100 mg/kg/day. ** *p *< 0.01 *vs*. C; ^## ^*p *< 0.01 *vs*. D based on one way ANOVA followed by Tukey's test.

Renal TNF-α and IL-1β protein expression assessed by Western blotting and were significantly increased in diabetic rats compared with those in control rats (*p *< 0.05), where as curcumin treatment significantly abrogated these increases in diabetic rats (*p *< 0.01) (Figure 3A,C,D). In line with protein analysis, renal TNF-α and IL-1β mRNA expression assessed by real-time PCR were also significantly higher in diabetic rats compared with those in control rats (*p *< 0.05), and curcumin treatment significantly attenuated the increased renal TNF-α and IL-1β mRNA expression (*p *< 0.05). The TNF-α/GAPDH mRNA and IL-1β/GAPDH mRNA ratios was 2.3 and 2.6 fold higher in diabetic rats compared with control rats, respectively, and curcumin treatment ameliorated these increases by 1.7 and 2.5 fold, respectively (Figure 4A,B).

### Effect of curcumin on renal ICAM-1 and TGF-β_1 _protein expression

**Figure 5:**
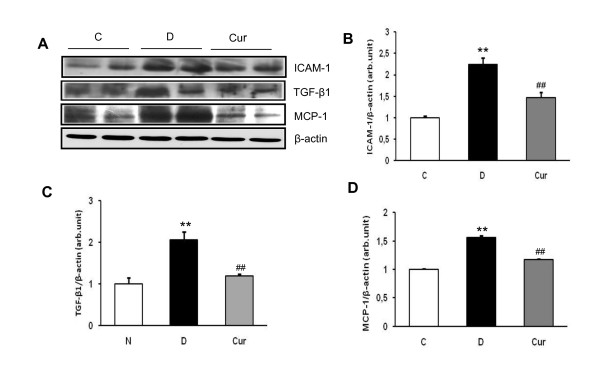
**Renal expressions of ICAM-1, MCP-1 and TGF-β_1_**. (A) Representative Western blots showing specific bands for ICAM-1, MCP-1, TGF-β_1_, and β-actin as an internal control. Equal amounts of protein sample obtained from whole kidney homogenates were applied in each lane. These bands are representative of five separate experiments. (B-D) Graphical representation of data from Western blots analyses. The mean density values of ICAM-1, MCP-1 and TGF-β_1 _are expressed as ratios relative to that of β-actin. Each bar represents mean ± SEM. C, age-matched normal rats; D, diabetic-treated rats administered with vehicle; Cur, diabetic rats treated with curcumin 100 mg/kg/day. ** *p *< 0.05 *vs*. C; ^## ^*p *< 0.05 *vs*. D based on one way ANOVA followed by Tukey's test.

Western blot analysis showed that the renal ICAM-1 protein expression was increased by 2.2 fold in the diabetic rats compared with that in the control rats (*p *< 0.05) and treatment with curcumin significantly decreased ICAM-1 protein levels in the diabetic kidney (*p *< 0.05) (Figure 5A,B). This finding was correlated with immunofluorescence staining, in which, ICAM-1 expression in renal tissues was significantly enhanced in diabetic rats compared with non-diabetic rats (Figure 2H), where as curcumin suppressed the increased intensity of ICAM-1 expression in the diabetic rats (Figure 2I). Western blot analysis also demonstrated a significant increase of renal TGF-β_1 _expression in the diabetic rats when compared with that in the control rats, which was significantly attenuated by treatment with curcumin (Figure 5A,B).

### Effect of curcumin on renal expression of MCP-1

MCP-1 is a chemokine that play an important role in the progression of DN. Using Western blot analysis we found that renal MCP-1 protein expression was increased by 1.5 fold in diabetic rats compared with that in the control rats (*p *< 0.05). This increase in renal MCP-1 protein expression was markedly suppressed by curcumin treatment in the diabetic rats (*p *< 0.05) (Figure 5A,D).

## Discussion

Macrophage accumulation and activation in the kidney have been shown to correlate with the onset of DN [4]. Using accumulation of ED-1 as a marker of macrophage activation, we have demonstrated increased activation of macrophage in the glomeruli of kidney tissue from diabetic animals. In this study, we demonstrate for the first time that macrophage infiltration in diabetic glomeruli is ameliorated by the administration of curcumin. In addition, the results of this study suggest that the anti-inflammatory effect of curcumin in DN is partly mediated by inhibiting ICAM-1 and MCP-1 expression under diabetic conditions. We also found that curcumin significantly suppressed NF-κB activity, a major transcriptional factor of many proinflammatory genes as well as reduced the degradation of cytosolic IκBα: as a consequence, the level of proinflammatory cytokines, TNF-α and IL-1β were further significantly decreased.

Curcumin is a highly pleiotropic molecule capable of interacting with numerous molecular targets involved in inflammation. Curcumin has been reported to modulate the inflammatory response by inhibiting the production of the inflammatory cytokines IL-8, monocyte inflammatory protein (MIP)-1α, MCP-1, IL-1β and TNF-α by monocyte and macrophage [25]. The beneficial effects of curcumin have also been demonstrated in several experimental kidney disease models [20,28-32]. Ghosh *et al. *[28] showed that curcumin treatment improved kidney function in animals with chronic renal failure by antagonizing the effect of TNF-α elicited NF-κB activation and macrophage infiltration, indicating that the anti-inflammatory property of curcumin may be responsible for alleviating chronic renal failure in nephrectomized animals. A recent study also demonstrated that curcumin treatment significantly reduced obesity induced inflammatory response and macrophage infiltration of white adipose tissue in murine models of insulin-resistant obesity and decreased hepatic NF-κB activity, an effect associated with decreased hepatic expression of inflammatory molecules [33,34]. Chiu *et al. *reported that curcumin prevents diabetes-associated abnormalities in the kidneys through the inhibition of NF-κB activation and p300 [20]. Curcumin is also known as an established inhibitor of NF-κB activation [35] and has recently been shown to specifically target inhibitory kappa B kinase (IκB) [19]. However, it is still unknown whether anti-inflammatory mechanism of curcumin through the inhibition of macrophage infiltration, might offer the renal protective effect in type I.

In recent years, several clinical and animal studies have indicated that inflammatory cytokines play an important roles in the development and progression of DN [36,37]. Schmid *et al. *have reported that upregulation of NF-κB targets, a master transcriptional gene play a role in inflammatory response in the kidney of the patients with progressive DN [38]. NF-κB is present in the cytoplasma complexed to its inhibitory protein known as IκB. After activation by a number of physiological and nonphysiological stimuli, such as IL-1β, TNF-α, or lipopolysaccharide, IκB dissociates from NF-κB within minutes and undergoes ubiquitination and degradation. Once NF-κB is released from the inhibitory unit IκB, the NF-κB is then translocated into the nucleus. Upon its nuclear translocation, NF-κB undergoes phosphorylation on serine 276 in its p65 subunit and associates with surrounding chromatin components. It subsequently binds with DNA and promotes the transcription of proinflammatory cytokines, such as TNF-α and IL-1β. Previous study has reported that phosphorylation on serine 276 is essential for NF-κB p65-dependent cellular responses [39]. Therefore, measurement of the phosphorylated p65 subunit of NF-κB is an effective tool for determining NF-κB activation [12,40]. Our results showed that activation of NF-κB and degradation of IκBα, as well as proinflammatory cytokines expression, TNF-α and IL-1β were increased in diabetic rats compared with the levels in control rats (Figures. 3 and 4). Curcumin treatment prevented all of these alterations. We believe that the curcumin's ability to inactivate NF-κB [41] and thus inhibited the production of proinflammatory cytokines [29] is its most likely mechanisms of action.

Macrophages are known to cause early glomerular injury in STZ-induced diabetes and also to be involved in the mechanisms that cause progressive glomerular and tubular damage [5,10,42,43]; in addition, ICAM-1 and MCP-1 are considered as a central molecule involved in macrophage influx in DN [9,11,44-46]. Park *et al. *have demonstrated that high glucose could upregulate ICAM-1 protein and mRNA expression in rat mesangial cells through the protein kinase C-NF-κB pathways [11,14]. ICAM-1 is also induced by inflammatory cytokines such as TNF-α, IL-1, and interferon-γ [47]. Recent studies provided evidences that both ICAM-1 gene deficiency [10,45] and anti-ICAM-1 monoclonal antibody [11] obviously inhibited the infiltration of monocytes/macrophages into the glomerulus and alleviated the extent of renal injury. Studies have also demonstrated that NF-κB was involved in the induction of MCP-1 in mesangial cell cultured under high glucose condition and subsequently mediated macrophage accumulation [9,48,49]. Consistent with these previous reports, we have also observed that ICAM-1 and MCP-1 expression were increased in rats with experimental DN (Figure 5), which was associated with marked macrophages infiltration (Figure 2), and that these increases under diabetic conditions were ameliorated by curcumin treatment. This effect might be mediated by curcumin's inhibition of NF-κB activation.

In the present study, diabetic glomerulosclerosis was ameliorated along with the inhibition of macrophage infiltration (Figure 2). Accumulating evidence suggests that, in DN, glomerulosclerosis is associated with TGF-β_1 _expression [50,51], which is related to macrophage infiltration in glomeruli [52]. TGF-β_1 _is assumed to mediate inflammatory response and exaggerate the progression of DN [53]. In vitro and in vivo studies have reported that macrophages stimulates mesangial cells to produce ECM proteins through TGF-β [44,54,55]. TGF-β can in turn induce ECM overproduction from mesangial cells in autocrine and paracrine fashions [45]. Previous study has revealed that infiltrated monocytes/macrophages release lysosomal enzymes, nitrous oxide, reactive oxygen intermediates and TGF-β, which have been reported to play an essential role in renal damage and the depletion of macrophage by irradiation decreased the gene expression of TGF-β and type IV collagen in the glomeruli of diabetic rats at 4 weeks after induction of diabetes, suggesting the pathological role of macrophages in the increased expression of ECM proteins [56,57]. From the above results it is obvious that macrophage infiltration is an important inducer of TGF-β_1_. The TGF-β_1 _contains a sequence located -715 to -707 bp where NF-κB binds to target TGF-β_1 _gene expression [58]. Thus, following NF-κB activation, marked infiltration of macrophages subsequently upregulated the TGF-β_1 _expression which further promotes the increased ECM synthesis in diabetes. Our results show that renal TGF-β_1 _protein expression was significantly increased in diabetic rats. Curcumin treatment obviously decreased renal TGF-β_1 _expression and this improvement was achieved most probably via the inhibition of NF-κB activation. Taken together, all the above results suggest that beneficial effect of curcumin in rats with DN is at least in part through inhibition of macrophage infiltration via inhibiting NF-κB mediated inflammatory response.

## Conclusion

We have shown that the curcumin treatment ameliorated DN in rat model of type I diabetes through the inhibition of macrophage infiltration in glomerulus due to its anti-inflammatory effect. In addition, our results support the findings that curcumin has a property to inhibit the activity of NF-κB as well as the degradation of IκBα and as a result decreased the expression of proinflammatory and profibrotic cytokines. Given these promising preclinical findings, we believe that the curcumin might be considered as potential adjuvant entity for preventing DN.

## Competing interests

The authors declare that they have no competing interests.

## Authors' contributions

VS and KW made substantial contributions to the conception and design of the study, analysis and interpretations of data, as well as gave final approval for the version to be published. FRS, PTV, and RAT participated in revising the manuscript. MH, APL, and HK, helped in the interpretation of data. VKS and KS participated in carrying out the RT-PCR. All authors contributed to the drafting of the manuscript and agreed on the final version of the manuscript.
